# Comprehensive Dementia Care to Address Multimorbidity Across Inpatient, Residential and Community Settings

**DOI:** 10.1093/geroni/igaf122.830

**Published:** 2025-12-31

**Authors:** Malaz Boustani

**Affiliations:** Indiana University, Indianapolis, Indiana, United States

## Abstract

Fully addressing dementia requires attention to patients at risk for dementia. The Aging Brain Care (ABC) program has been addressing this along with its sustainability and scalability over the past 17 years. The ABC populations disproportionately suffer from dementia, comorbid medical conditions, and high social frailty. To address comorbidities, the ABC program conducts a comprehensive assessment to diagnose dementia and its severity, reviews medical conditions, assesses the burden on informal caregivers, determines the biopsychosocial needs of the dyads, and identify their goals and preferences. Then it co-develops a personalized care plan including de-prescribing inappropriate medications and doing co management of comorbid conditions in collaboration with primary care. In a clinical trial, conducted in Federally Qualified Health Centers, the ABC program reduced the behavioral and psychological symptoms related to dementia, the burden of informal caregivers, and the use of inappropriate medications. To enhance the ABC program’s work with vulnerable populations, it facilitates care transition from home to hospital and subacute rehabilitation and back to home. ABC also partners with community-based organizations to identify home and community-based resources to overcome care needs and address social determinants of health. Community health workers trained and supported to deliver care across settings in accordance with the patients’ and family preference have provided care to an average 400 dyads per year. Work is now underway to expand the care navigation roles to address conditions other than dementia, and to support the diagnostic process for patients at risk for dementia.

